# Supplementary material for: A novel pathogenic avipoxvirus infecting oriental turtle dove (Streptopelia orientalis) in China shows a high genomic and evolutionary proximity with the pigeon avipoxviruses isolated globally
Source: Microbiol Spectr. 2023 Sep 26;11(5):e01193-23. doi: 10.1128/spectrum.01193-23 (PMC10581063; doi:10.1128/spectrum.01193-23)
Supplement: Supplemental material — Fig. S1 to S4; Table S1. [file spectrum.01193-23-s0001.pdf]

Supplementary File

A Novel Pathogenic Avipoxvirus Infecting Oriental Turtle Dove (*Streptopelia orientalis*) in China Shows a High Genomic and Evolutionary Proximity with the Pigeon Avipoxviruses Isolated Globally

Lei He[1,*,#], Yuhao Zhang[1,#], Yanyan Jia[1], Zedian Li[1], Jing Li[1], Ke Shang[1], Ke Ding[1], Haotong Yu[1], Subir Sarker[2]*

1.The Key Lab of Animal Disease and Public Health /Luoyang Key Laboratory of Live Carrier Biomaterial and Animal Disease Prevention and Control, Henan University of Science and Technology, Luoyang 471023, Henan, China;

2. Department of Microbiology, Anatomy, Physiology and Pharmacology, School of Agriculture, Biomedicine and Environment, La Trobe University, Melbourne, VIC 3086, Australia

Running title: Novel pathogenic avipoxvirus infection in oriental turtle dove.

* Corresponding author Lei He. The Key Lab of Animal Disease and Public Health /Luoyang Key Laboratory of Live Carrier Biomaterial and Animal Disease Prevention and Control, Henan University of Science and Technology, Luoyang 471023, Henan, China; Tel: +15236269770, E-mail: helei4280546@163.com

* Corresponding author Subir Sarker. Department of Microbiology, Anatomy,

Physiology and Pharmacology, School of Agriculture, Biomedicine and Environment, La Trobe University, Melbourne, VIC 3086, Australia. E-mail: s.sarker@latrobe.edu.au.

# = contributed equally to this work.

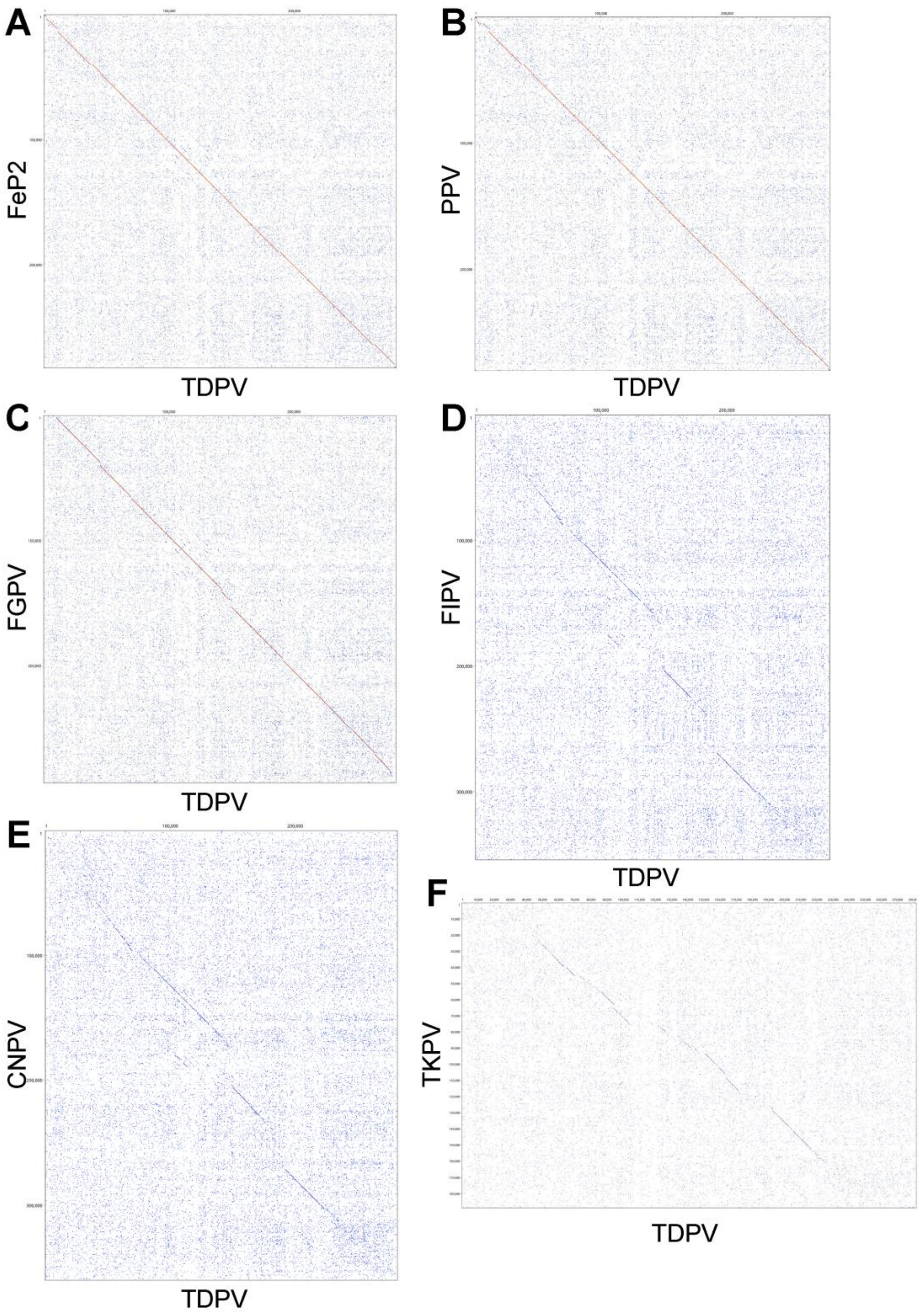

FIG S1 Dot plots of the ALPV genome (x-axis) vs. other poxvirus genomes (y-axis). (A) TDPV vs. FeP2, (B) TDPV vs. PPV, (C) TDPV vs. FGPV, (D) TDPV vs. FIPV, (E) TDPV vs. CNPV and (F) TDPV vs. TKPV (refer to Table 1 for virus details and GenBank accession numbers). The Classic colour scheme was chosen in Geneious

Prime software (version 2022.2.2) for the dot plot lines according to the length of the match, from blue for short matches to red for matches over 100 bp long.

	ALPV	ALPV2	ChePV1	CNPV	CPPV	CRPV	FeP2	FGPV	FIPV	FWPV	FWPV	FWPV	FWPV	FWPV	FWPV	FWPV	FWPV	FWPV	FWPV	MLPV	MPPV	MPPV2	PEPV	PEPV2	PPV	SwCRV1	SWPV1	SWPV2	TDPV	TePV1	TKPV
ALPV		82.1%	91.2%	99.9%	82.1%	98.7%	82.2%	82.1%	89.9%	82.1%	82.1%	82.1%	82.0%	82.0%	82.0%	82.0%	82.1%	82.1%	82.1%	99.0%	98.9%	98.9%	82.2%	100%	82.2%	51.8%	90.3%	100%	82.1%	78.3%	78.6%
ALPV2	82.1%		82.0%	82.1%	97.1%	82.0%	96.2%	97.1%	75.3%	100.0%	99.8%	99.9%	99.9%	99.8%	99.8%	99.8%	99.9%	99.9%	99.8%	82.0%	81.9%	82.0%	96.5%	82.1%	96.2%	52.1%	81.7%	82.1%	96.2%	80.7%	80.8%
ChePV1	91.2%	82.0%		91.2%	82.0%	91.1%	81.9%	82.0%	83.5%	82.1%	82.1%	82.0%	82.0%	82.0%	82.0%	82.0%	82.1%	82.1%	82.1%	92.2%	91.2%	91.0%	82.1%	91.2%	81.9%	52.0%	91.5%	91.2%	81.9%	78.7%	79.0%
CNPV	99.9%	82.1%	91.2%		82.2%	98.7%	82.2%	82.2%	90.0%	82.1%	82.2%	82.1%	82.1%	82.1%	82.1%	82.1%	82.2%	82.2%	82.2%	98.9%	98.8%	98.9%	82.3%	99.9%	82.2%	51.9%	90.3%	99.9%	82.2%	78.4%	78.6%
CPPV	82.1%	97.1%	82.0%	82.2%		82.1%	98.5%	99.5%	75.4%	97.1%	97.2%	97.2%	97.1%	97.1%	97.1%	97.1%	97.2%	97.2%	97.2%	82.1%	82.0%	82.0%	98.8%	82.1%	98.5%	52.1%	81.7%	82.1%	98.4%	80.8%	80.8%
CRPV	98.7%	82.0%	91.1%	98.7%	82.1%		82.1%	82.0%	89.4%	82.0%	82.1%	82.0%	82.0%	82.0%	82.0%	82.0%	82.1%	82.1%	82.1%	97.9%	98.5%	98.7%	82.2%	98.7%	82.1%	51.8%	90.1%	98.7%	82.0%	78.3%	78.6%
FeP2	82.2%	96.2%	81.9%	82.2%	98.5%	82.1%		98.4%	75.4%	96.3%	96.3%	96.3%	96.3%	96.2%	96.3%	96.3%	96.3%	96.3%	96.3%	82.1%	82.0%	82.0%	98.6%	82.2%	100%	52.2%	81.7%	82.2%	99.2%	80.6%	80.7%
FGPV	82.1%	97.1%	82.0%	82.2%	99.5%	82.0%	98.4%		75.4%	97.1%	97.1%	97.1%	97.1%	97.0%	97.1%	97.1%	97.1%	97.1%	97.2%	82.0%	82.0%	82.0%	98.7%	82.1%	98.4%	52.2%	81.7%	82.1%	98.3%	80.7%	80.9%
FIPV	89.9%	75.3%	83.5%	90.0%	75.4%	89.4%	75.4%	75.4%		75.3%	75.3%	75.3%	75.3%	75.3%	75.3%	75.3%	75.3%	75.3%	75.4%	90.0%	89.4%	89.3%	75.5%	89.9%	75.4%	47.8%	82.6%	89.9%	75.3%	72.2%	71.8%
FWPV	82.1%	100.0%	82.1%	82.1%	97.1%	82.0%	96.3%	97.1%	75.3%		99.8%	99.9%	99.9%	99.8%	99.9%	99.9%	99.9%	99.9%	99.8%	82.0%	82.0%	82.0%	96.5%	82.1%	96.3%	52.1%	81.7%	82.1%	96.2%	80.7%	80.8%
FWPV	82.1%	99.8%	82.1%	82.2%	97.2%	82.1%	96.3%	97.1%	75.3%	99.8%		99.8%	99.8%	99.7%	99.8%	99.8%	99.9%	99.9%	99.9%	82.1%	82.0%	82.0%	96.6%	82.1%	96.3%	52.2%	81.7%	82.1%	96.2%	80.8%	80.9%
FWPV	82.1%	99.9%	82.0%	82.1%	97.2%	82.0%	96.3%	97.1%	75.3%	99.9%	99.8%		99.9%	99.9%	99.9%	99.9%	100.0%	100.0%	99.8%	82.0%	82.0%	82.0%	96.6%	82.1%	96.3%	52.1%	81.7%	82.1%	96.2%	80.7%	80.8%
FWPV	82.0%	99.9%	82.0%	82.1%	97.1%	82.0%	96.3%	97.1%	75.3%	99.9%	99.8%	99.9%		99.8%	99.8%	99.8%	99.9%	99.9%	99.8%	82.0%	81.9%	81.9%	96.5%	82.0%	96.3%	52.1%	81.7%	82.0%	96.2%	80.7%	80.8%
FWPV	82.0%	99.8%	82.0%	82.1%	97.1%	82.0%	96.2%	97.0%	75.3%	99.8%	99.7%	99.9%	99.8%		99.8%	99.8%	99.9%	99.9%	99.8%	82.0%	81.9%	81.9%	96.5%	82.0%	96.2%	52.1%	81.6%	82.0%	96.1%	80.6%	80.8%
FWPV	82.0%	99.8%	82.0%	82.1%	97.1%	82.0%	96.3%	97.1%	75.3%	99.9%	99.8%	99.9%	99.8%	99.8%		100%	99.9%	99.9%	99.9%	82.0%	81.9%	81.9%	96.5%	82.0%	96.3%	52.2%	81.7%	82.0%	96.2%	80.7%	80.8%
FWPV	82.0%	99.8%	82.0%	82.1%	97.1%	82.0%	96.3%	97.1%	75.3%	99.9%	99.8%	99.9%	99.8%	99.8%	100%		99.9%	99.9%	99.9%	82.0%	81.9%	81.9%	96.5%	82.0%	96.3%	52.2%	81.7%	82.0%	96.2%	80.7%	80.8%
FWPV	82.1%	99.9%	82.1%	82.2%	97.2%	82.1%	96.3%	97.1%	75.3%	99.9%	99.9%	100.0%	99.9%	99.9%	99.9%	99.9%		100%	99.9%	82.1%	82.0%	82.0%	96.6%	82.1%	96.3%	52.2%	81.7%	82.1%	96.2%	80.7%	80.9%
FWPV	82.1%	99.9%	82.1%	82.2%	97.2%	82.1%	96.3%	97.1%	75.3%	99.9%	99.9%	100.0%	99.9%	99.9%	99.9%	99.9%	100%		99.9%	82.1%	82.0%	82.0%	96.6%	82.1%	96.3%	52.2%	81.7%	82.1%	96.2%	80.7%	80.9%
FWPV	82.1%	99.8%	82.1%	82.2%	97.2%	82.1%	96.3%	97.2%	75.4%	99.8%	99.9%	99.8%	99.8%	99.8%	99.9%	99.9%	99.9%	99.9%		82.1%	82.0%	82.0%	96.6%	82.1%	96.3%	52.2%	81.8%	82.1%	96.3%	80.8%	80.9%
MLPV	99.0%	82.0%	92.2%	98.9%	82.1%	97.9%	82.1%	82.0%	90.0%	82.0%	82.1%	82.0%	82.0%	82.0%	82.0%	82.0%	82.1%	82.1%	82.1%		98.2%	98.0%	82.1%	99.0%	82.1%	51.8%	90.5%	99.0%	82.0%	78.4%	78.6%
MPPV	98.9%	81.9%	91.2%	98.8%	82.0%	98.5%	82.0%	82.0%	89.4%	82.0%	82.0%	82.0%	81.9%	81.9%	81.9%	81.9%	82.0%	82.0%	82.0%	98.2%		99.6%	82.1%	98.9%	82.0%	51.8%	90.0%	98.9%	82.0%	78.3%	78.6%
MPPV2	98.9%	82.0%	91.0%	98.9%	82.0%	98.7%	82.0%	82.0%	89.3%	82.0%	82.0%	82.0%	81.9%	81.9%	81.9%	81.9%	82.0%	82.0%	82.0%	98.0%	99.6%		82.1%	98.9%	82.0%	51.7%	89.9%	98.9%	82.0%	78.2%	78.5%
PEPV	82.2%	96.5%	82.1%	82.3%	98.8%	82.2%	98.6%	98.7%	75.5%	96.5%	96.6%	96.6%	96.5%	96.5%	96.5%	96.5%	96.6%	96.6%	96.6%	82.1%	82.1%	82.1%		82.2%	98.6%	52.1%	81.9%	82.2%	98.5%	80.7%	80.8%
PEPV2	100%	82.1%	91.2%	99.9%	82.1%	98.7%	82.2%	82.1%	89.9%	82.1%	82.1%	82.1%	82.0%	82.0%	82.0%	82.0%	82.1%	82.1%	82.1%	99.0%	98.9%	98.9%	82.2%		82.2%	51.8%	90.3%	100%	82.1%	78.3%	78.6%
PPV	82.2%	96.2%	81.9%	82.2%	98.5%	82.1%	100%	98.4%	75.4%	96.3%	96.3%	96.3%	96.3%	96.2%	96.3%	96.3%	96.3%	96.3%	96.3%	82.1%	82.0%	82.0%	98.6%	82.2%		52.2%	81.7%	82.2%	99.2%	80.6%	80.7%
SwCRV1	51.8%	52.1%	52.0%	51.9%	52.1%	51.8%	52.2%	52.2%	47.8%	52.1%	52.2%	52.1%	52.1%	52.1%	52.2%	52.2%	52.2%	52.2%	52.2%	51.8%	51.8%	51.7%	52.1%	51.8%	52.2%		51.9%	51.8%	52.1%	51.7%	52.1%
SWPV1	90.3%	81.7%	91.5%	90.3%	81.7%	90.1%	81.7%	81.7%	82.6%	81.7%	81.7%	81.7%	81.7%	81.6%	81.7%	81.7%	81.7%	81.7%	81.8%	90.5%	90.0%	89.9%	81.9%	90.3%	81.7%	51.9%		90.3%	81.6%	78.5%	78.8%
SWPV2	100%	82.1%	91.2%	99.9%	82.1%	98.7%	82.2%	82.1%	89.9%	82.1%	82.1%	82.1%	82.0%	82.0%	82.0%	82.0%	82.1%	82.1%	82.1%	99.0%	98.9%	98.9%	82.2%	100%	82.2%	51.8%	90.3%		82.1%	78.3%	78.6%
TDPV	82.1%	96.2%	81.9%	82.2%	98.4%	82.0%	99.2%	98.3%	75.3%	96.2%	96.2%	96.2%	96.2%	96.1%	96.2%	96.2%	96.2%	96.2%	96.3%	82.0%	82.0%	82.0%	98.5%	82.1%	99.2%	52.1%	81.6%	82.1%		80.5%	80.5%
TePV1	78.3%	80.7%	78.7%	78.4%	80.8%	78.3%	80.6%	80.7%	72.2%	80.7%	80.8%	80.7%	80.7%	80.6%	80.7%	80.7%	80.7%	80.7%	80.8%	78.4%	78.3%	78.2%	80.7%	78.3%	80.6%	51.7%	78.5%	78.3%	80.5%		77.8%
TKPV	78.6%	80.8%	79.0%	78.6%	80.8%	78.6%	80.7%	80.9%	71.8%	80.8%	80.9%	80.8%	80.8%	80.8%	80.8%	80.8%	80.9%	80.9%	80.9%	78.6%	78.6%	78.5%	80.8%	78.6%	80.7%	52.1%	78.8%	78.6%	80.5%	77.8%	

FIG S2 The matrix illustration pairwise comparison among the concatenated amino acid sequences of the selected nine poxvirus core proteins using Geneious software (Version 2022.2.2). The gradient from black to white indicates a high to low homology between the two sequences. Details of the poxviruses used in the matrix illustration can be seen in Table 2.

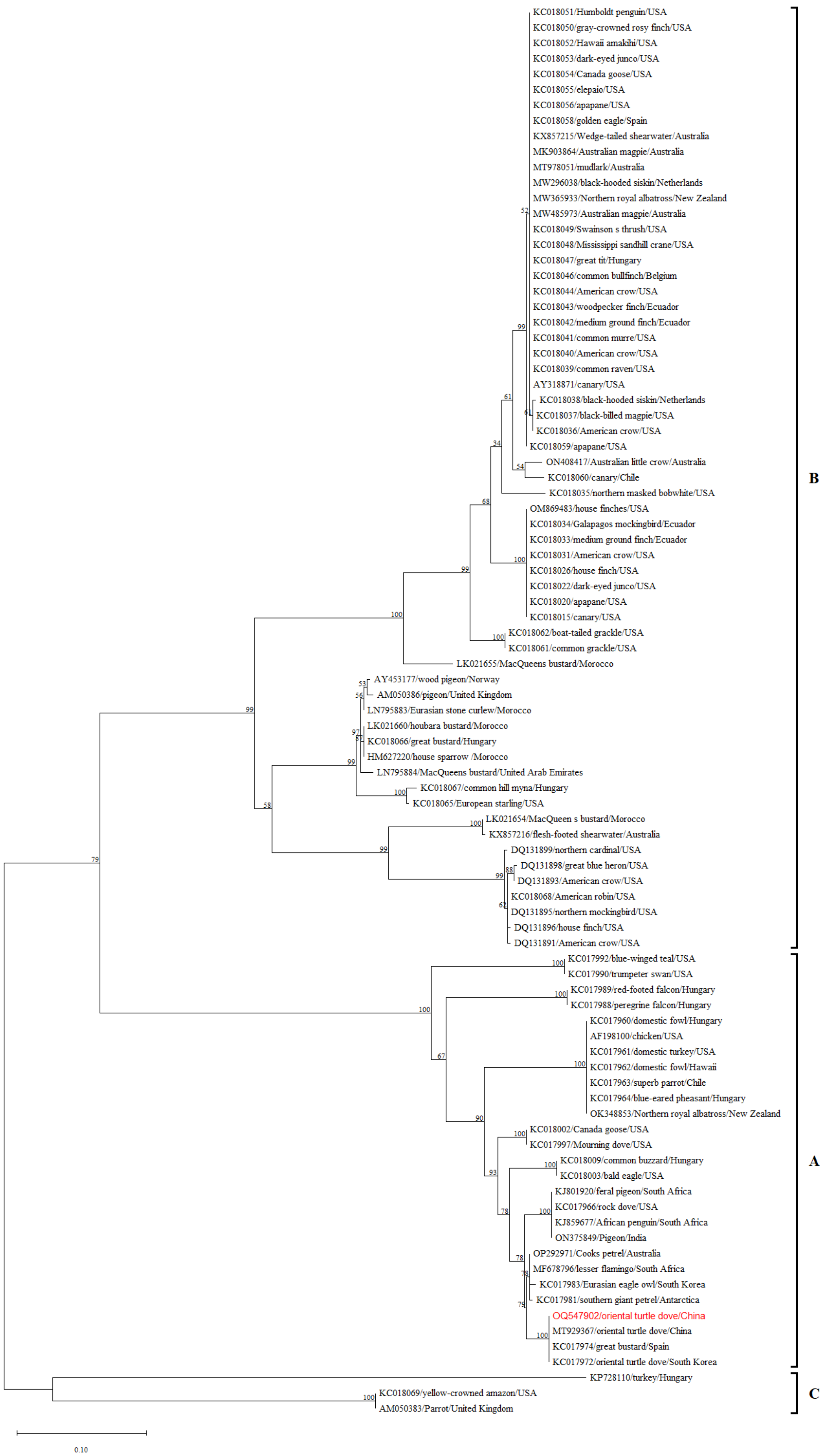

KC018051/Humboldt penguin/USA
KC018050/gray-crowned rosy finch/USA
KC018052/Hawaii amakihi/USA
KC018053/dark-eyed junco/USA
KC018054/Canada goose/USA
KC018055/elepaio/USA
KC018056/apapane/USA
KC018058/golden eagle/Spain
KX857215/Wedge-tailed shearwater/Australia
MK903864/Australian magpie/Australia
MT978051/mudlark/Australia
MW296038/black-hooded siskin/Netherlands
MW365933/Northern royal albatross/New Zealand
MW485973/Australian magpie/Australia
KC018049/Swainson s thrush/USA
KC018048/Mississippi sandhill crane/USA
KC018047/great tit/Hungary
KC018046/common bullfinch/Belgium
KC018044/American crow/USA
KC018043/woodpecker finch/Ecuador
KC018042/medium ground finch/Ecuador
KC018041/common murre/USA
KC018040/American crow/USA
KC018039/common raven/USA
AY318871/canary/USA
KC018038/black-hooded siskin/Netherlands
KC018037/black-billed magpie/USA
KC018036/American crow/USA
KC018059/apapane/USA
ON408417/Australian little crow/Australia
KC018060/canary/Chile
KC018035/northern masked bobwhite/USA
OM869483/house finches/USA
KC018034/Galapagos mockingbird/Ecuador
KC018033/medium ground finch/Ecuador
KC018031/American crow/USA
KC018026/house finch/USA
KC018022/dark-eyed junco/USA
KC018020/apapane/USA
KC018015/canary/USA
KC018062/boat-tailed grackle/USA
KC018061/common grackle/USA
LK021655/MacQueens bustard/Morocco
AY453177/wood pigeon/Norway
AM050386/pigeon/United Kingdom
LN795883/Eurasian stone curlew/Morocco
LK021660/houbara bustard/Morocco
KC018066/great bustard/Hungary
HM627220/house sparrow /Morocco
LN795884/MacQueens bustard/United Arab Emirates
KC018067/common hill myna/Hungary
KC018065/European starling/USA
LK021654/MacQueen s bustard/Morocco
KX857216/flesh-footed shearwater/Australia
DQ131899/northern cardinal/USA
DQ131898/great blue heron/USA
DQ131893/American crow/USA
KC018068/American robin/USA
DQ131895/northern mockingbird/USA
DQ131896/house finch/USA
DQ131891/American crow/USA
KC017992/blue-winged teal/USA
KC017990/trumpeter swan/USA
KC017989/red-footed falcon/Hungary
KC017988/peregrine falcon/Hungary
KC017960/domestic fowl/Hungary
AF198100/chicken/USA
KC017961/domestic turkey/USA
KC017962/domestic fowl/Hawaii
KC017963/superb parrot/Chile
KC017964/blue-eared pheasant/Hungary
OK348853/Northern royal albatross/New Zealand
KC018002/Canada goose/USA
KC017997/Mourning dove/USA
KC018009/common buzzard/Hungary
KC018003/bald eagle/USA
KJ801920/feral pigeon/South Africa
KC017966/rock dove/USA
KJ859677/African penguin/South Africa
ON375849/Pigeon/India
OP292971/Cooks petrel/Australia
MF678796/lesser flamingo/South Africa
KC017983/Eurasian eagle owl/South Korea
KC017981/southern giant petrel/Antarctica
OQ547902/oriental turtle dove/China
MT929367/oriental turtle dove/China
KC017974/great bustard/Spain
KC017972/oriental turtle dove/South Korea
KP728110/turkey/Hungary
KC018069/yellow-crowned amazon/USA
AM050383/Parrot/United Kingdom
B
A
C
0.10

FIG S3 A maximum likelihood (ML) tree was constructed from multiple alignments of the partial nucleotide sequences of the P4b gene of selected avipoxviruses using MEGA software (Version 11.0.11) with 1000 bootstraps. The numbers on the left show bootstrap values as percentages (0-100). Labels at branch tips refer to GenBank accession number/species/country of origin. The position of novel TDPV is highlighted using red text and the major clades is designated according to Gyuranecz et al.(3).

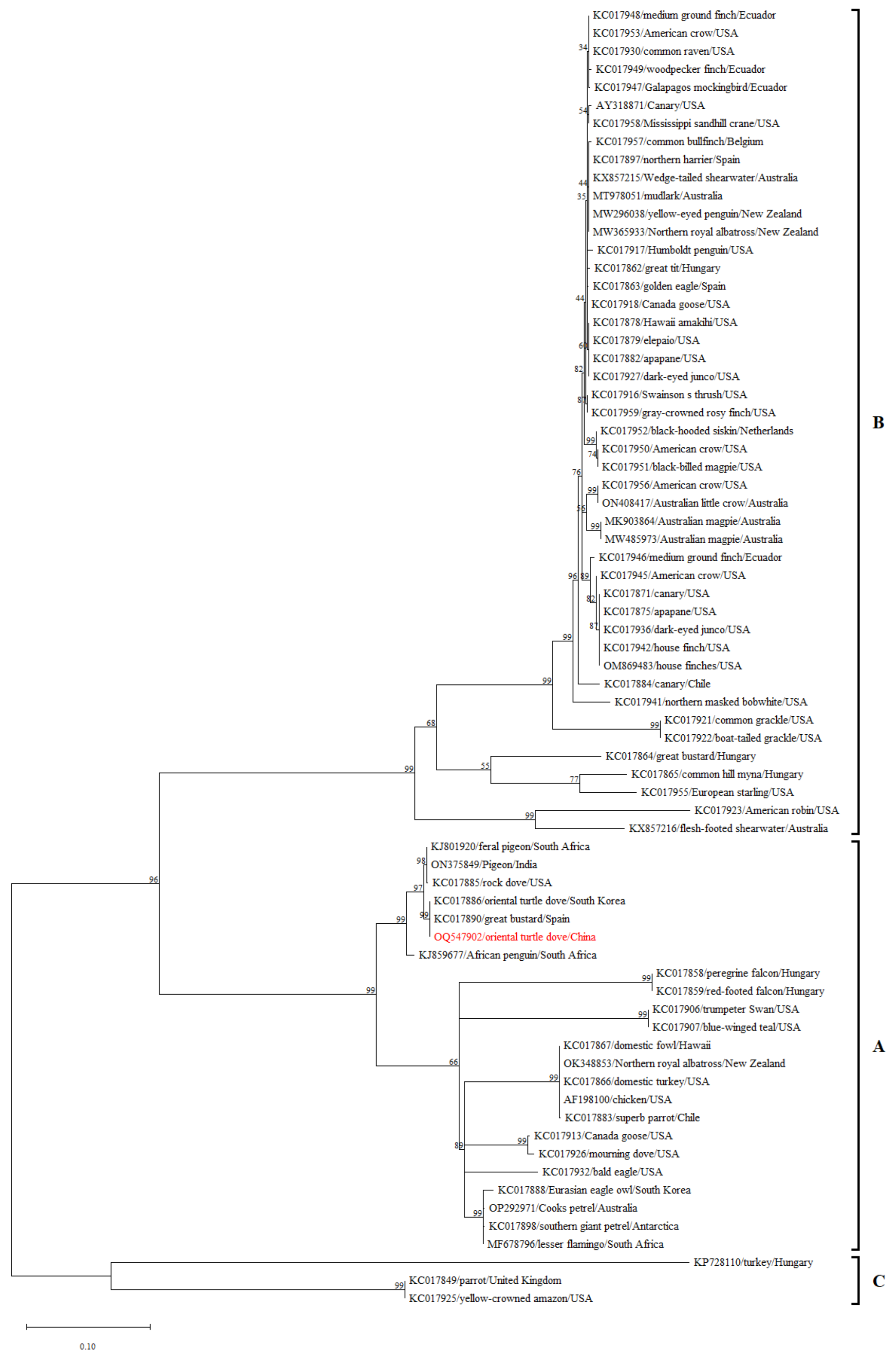

FIG S4 A maximum likelihood (ML) tree was constructed from multiple alignments of the partial nucleotide sequences of the DNA polymerase gene of selected avipoxviruses

using MEGA software (Version 11.0.11) with 1000 bootstraps. The numbers on the left show bootstrap values as percentages (0-100). Labels at branch tips refer to GenBank accession number/species/country of origin. The position of novel TDPV is highlighted using red text and the major clades is designated according to Gyuranecz et al.(3).

Supplementary TABLE S1 Oriental turtle dovepox virus (TDPV) genome annotations and comparative analysis of ORFs.

TDPV Synteny	TDPV-Genome Coordinates	TDPV AA Size	FeP2 Synteny	FeP2 AA Size	Best Blast hits	Product	TDPV Identity (%) compared to avipoxviruses AA identity	Notes
TDPV-001	198-97	33			unique			hypothetical protein, unique to TDPV
TDPV-002	475-287	62			unique			hypothetical protein, unique to TDPV
TDPV-003	1221-616	201	FeP2 001	200	FeP2 001	C-type lectin family protein	91.54%	I
TDPV-004	1746-1612	44			unique			hypothetical protein, unique to TDPV
TDPV-005	1933-2601	222	FeP2 003	222	PEPV 004	hypothetical protein	95.50%	I
TDPV-006	2619-2834	71			CPPV 006	hypothetical protein	91.55%	F/T
TDPV-007	2819-3064	81			CPPV 006	hypothetical protein	91.53%	F/T
TDPV-008	3925-3350	191	FeP2 258	192	PEPV 006	ankyrin repeat family protein	92.15%	I
TDPV-009	4852-4944	30			unique			hypothetical protein, unique to TDPV
TDPV-010	6363-4957	468	FeP2 006	467	PEPV 279	Ig-like domain protein	97.65%	F/T
TDPV-011	6497-6402	31			unique			hypothetical protein, unique to TDPV, containing a transmembrane helix
TDPV-012	6618-6818	66			FWPV 004	hypothetical protein	97.65%	F/T
TDPV-013	7242-7105	45			CPPV 010	hypothetical protein	81.82%	I
TDPV-014	7177-7338	53			unique			hypothetical protein, unique to TDPV
TDPV-015	7646-7461	61	FeP2 007	78	PEPV 006	ankyrin repeat protein	88.52%	F/T
TDPV-016	7987-7781	68	FeP2 258	192	CPPV 011	ankyrin repeat protein	86.57%	F/T
TDPV-017	8206-9471	421	FeP2 008	410	FGPV 284	C4L/C10L-like family protein	94.30%	F/T
TDPV-018	10000-10104	34			FGPV 001	ankyrin repeat family protein	72.73%	F/T
TDPV-019	10290-10472	60			FGPV 001	ankyrin repeat family protein	57.33%	F/T
TDPV-020	10472-10585	37			FGPV 001	ankyrin repeat family protein	71.11%	F/T
TDPV-021	11713-10580	377	FeP2 011	680	FeP2 011	ankyrin repeat protein	95.44%	F/T
TDPV-022	12101-11685	138	FeP2 011	680	FeP2 011	ankyrin repeat protein	97.06%	F/T
TDPV-023	12082-12309	75			unique			hypothetical protein, unique to TDPV
TDPV-024	12455-12288	55	FeP2 011	680	FeP2 011	ankyrin repeat protein	97.78%	F/T
TDPV-025	12528-12418	36	FeP2 011	680	FeP2 011	ankyrin repeat protein	91.18%	F/T
TDPV-026	13701-12634	355	FeP2 012	355	FeP2 012	serpin family protein	98.59%	I
TDPV-027	14688-13807	293	FeP2 013	293	FeP2 013	alphaSNAP	97.95%	I
TDPV-028	16320-14764	518	FeP2 014	518	PPV 013	hypothetical protein	97.88%	I
TDPV-029	16990-16454	178	FeP2 015	178	FeP2 015	interleukin 10	94.38%	I
TDPV-030	18042-17050	330	FeP2 016	330	PPV 015	ankyrin repeat protein	97.27%	I
TDPV-031	19354-18143	403	FeP2 017	403	FeP2 017	ankyrin repeat protein	94.79%	I
TDPV-032	20751-19438	437	FeP2 018	437	FeP2 018	ankyrin repeat protein	97.71%	I

TDPV-033	20750-20878	42			CPPV 033	hypothetical protein	83.33%	F/T
TDPV-034	21008-20868	46			CPPV 034	hypothetical protein	97.83%	I
TDPV-035	20991-21092	33			unique			hypothetical protein, unique to TDPV
TDPV-036	21643-21134	169	FeP2 019	170	FeP2 019	hypothetical protein	97.65%	I
TDPV-037	22616-21903	237	FeP2 020	237	FeP2 020	hypothetical protein	97.47%	I
TDPV-038	23466-22729	245	FeP2 021	245	FeP2 021	V-type Ig domain protein	96.33%	I
TDPV-039	25578-23527	683	FeP2 022	683	FeP2 022	ankyrin repeat protein	98.39%	I
TDPV-040	26047-25706	113	FeP2 023	97	FeP2 023	hypothetical protein	95.74%	E
TDPV-041	26076-26645	189	FeP2 024	189	FeP2 024	hypothetical protein	97.35%	I
TDPV-042	27925-26642	427	FeP2 025	427	FeP2 025	C4L|C10L-like family protein	98.55%	I
TDPV-043	28019-29014	331	FeP2 026	331	FeP2 026	G-protein-coupled receptor family protein	97.89%	I
TDPV-044	30774-29029	581	FeP2 027	581	FeP2 027	ankyrin repeat protein	97.42%	I
TDPV-045	30796-30888	30			CPPV 045	hypothetical protein	100.00%	I
TDPV-046	32154-30850	434	FeP2 028	434	CPPV 046	ankyrin repeat protein	97.00%	I
TDPV-047	34005-32221	594	FeP2 029	594	FeP2 029	ankyrin repeat protein	98.48%	I
TDPV-048	34746-34135	203	FeP2 030	203	FeP2 030	hypothetical protein	98.52%	I
TDPV-049	36305-34809	498	FeP2 031	498	PPV 030	ankyrin repeat protein	95.38%	I
TDPV-050	36426-36539	37			FGPV 024	ankyrin repeat protein	80.00%	F/T
TDPV-051	36770-36558	70	FeP2 032	67	FGPV 024	ankyrin repeat protein	85.29%	F/T
TDPV-052	36850-37134	94			FGPV 024	ankyrin repeat protein	51.02%	F/T
TDPV-053	37341-37457	38			unique			hypothetical protein, unique to TDPV
TDPV-054	37540-38547	335			FGPV 025	G-protein-coupled receptor family protein	93.73%	I
TDPV-055	38692-38582	36			FGPV 026	hypothetical protein	78.12%	F/T
TDPV-056	38802-38903	33			FGPV 026	hypothetical protein	96.88%	F/T
TDPV-057	40156-39797	119	FeP2 034	464	FeP2 034	ankyrin repeat protein	86.73%	F/T
TDPV-058	40480-40295	61	FeP2 034	464	FeP2 034	ankyrin repeat protein	98.36%	F/T
TDPV-059	40924-40544	126	FeP2 035	126	FeP2 035	hypothetical protein	100.00%	I
TDPV-060	43455-41029	808	FeP2 036	808	FeP2 036	alkaline phosphodiesterase	98.14%	I
TDPV-061	44607-43582	341	FeP2 037	341	FeP2 037	ankyrin repeat protein	97.07%	I
TDPV-062	44838-44945	35			unique			hypothetical protein, unique to TDPV
TDPV-063	45489-45340	49			FGPV 033	ankyrin repeat protein	63.27%	F/T
TDPV-064	46039-45632	135	FeP2 039	135	FeP2 039	hypothetical protein	97.78%	I
TDPV-065	46889-46395	164	FeP2 040	164	FeP2 040	hypothetical protein	98.78%	I
TDPV-066	47323-46886	145	FeP2 041	145	FGPV 037	dUTP pyrophosphatase	98.62%	I
TDPV-067	47902-47375	175	FeP2 042	175	FeP2 042	Bcl-2	96.00%	I
TDPV-068	48963-47950	337	FeP2 043	337	FeP2 043	serpin family protein	98.45%	I
TDPV-069	49684-49046	212	FeP2 044	226	CPPV 069	hypothetical protein	92.92%	I
TDPV-070	51465-49771	564	FeP2 045	564	FeP2 045	DNA ligase	97.51%	I

TDPV-071	52576-51500	358	FeP2 046	358	FeP2 046	serpin family protein	98.88%	I
TDPV-072	53730-52618	370	FeP2 047	370	FeP2 047	hydroxysteroid dehydrogenase	97.84%	I
TDPV-073	54992-53886	368	FeP2 048	576	CPPV 074	semaphorin	95.38%	F/T
TDPV-074	55615-55055	186	FeP2 048	576	FeP2 048	semaphorin	96.77%	F/T
TDPV-075	55941-55729	70			CPPV 076	hypothetical protein	85.71%	I
TDPV-076	56036-56821	261	FeP2 049	261	FeP2 049	GNS1|SUR4	99.62%	I
TDPV-077	56898-57362	154	FeP2 050	154	FeP2 050	late transcription factor VLTF2	100.00%	I
TDPV-078	57384-59042	552	FeP2 051	552	FeP2 051	rifampicin resistance N3L protein	99.63%	I
TDPV-079	59074-59943	289	FeP2 052	289	FeP2 052	mRNA capping enzyme	99.65%	I
TDPV-080	60020-61933	637	FeP2 053	637	FeP2 053	NPH-1 transcription termination factor	98.43%	I
TDPV-081	62618-61941	225	FeP2 054	225	FeP2 054	muT motif expression regulator	98.67%	I
TDPV-082	63294-62596	232	FeP2 055	237	FeP2 055	muT motif	97.85%	I
TDPV-083	64385-63561	274	FeP2 056	274	PEPV 057	V-type Ig domain protein	93.80%	I
TDPV-084	65036-64551	161	FeP2 057	161	FeP2 057	RNA polymerase subunit RPO18	98.76%	I
TDPV-085	66924-65023	633	FeP2 058	633	FeP2 058	early transcription factor VETF	99.53%	I
TDPV-086	69280-66905	791	FeP2 059	791	FeP2 059	NTPase	99.49%	I
TDPV-087	69745-69858	37			CPPV 090	CC chemokine-like protein	57.89%	F/T
TDPV-088	69834-70037	67			CPPV 090	CC chemokine-like protein	66.10%	F/T
TDPV-089	70218-70054	54			unique			hypothetical protein, unique to TDPV
TDPV-090	70402-70515	37			unique			hypothetical protein, unique to TDPV
TDPV-091	70799-70948	49			FGPV 057	CC-chemokine family protein	47.83%	F/T
TDPV-092	71595-70993	200	FeP2 060	200	PPV 059	CC chemokine family protein	97.50%	I
TDPV-093	71858-71718	46			unique			hypothetical protein, unique to TDPV
TDPV-094	71963-71862	33			unique			hypothetical protein, unique to TDPV
TDPV-095	72479-72150	109	FeP2 061	109	FeP2 061	CC chemokine family protein	98.17%	I
TDPV-096	73158-72559	199	FeP2 062	199	FeP2 062	CC chemokine family protein	92.46%	I
TDPV-097	73860-73204	218	FeP2 063	218	FeP2 063	uracil DNA glycosylase	100.00%	I
TDPV-098	75113-73911	400	FeP2 065	196	FGPV 062	hypothetical protein	91.73%	I
TDPV-099	75264-75154	36			unique			hypothetical protein, unique to TDPV
TDPV-100	75241-75369	42			CPPV 102	phospholipid hydroperoxide glutathione peroxidase-like proteinue	97.62%	I
TDPV-101	75442-75846	134	FeP2 066	134	CPPV 103	phospholipid hydroperoxide glutathione peroxidase-like proteinue	98.51%	I
TDPV-102	75847-76179	110	FeP2 067	110	FeP2 067	hypothetical protein	98.18%	I
TDPV-103	76570-76154	138	FeP2 068	138	FeP2 068	hypothetical protein	98.55%	I
TDPV-104	76925-76668	85	FeP2 069	93	FeP2 069	HT motif family protein	98.82%	F/T
TDPV-105	77705-77313	130	FeP2 070	131	FeP2 070	hypothetical protein	96.52%	I
TDPV-106	77704-77805	33			unique			hypothetical protein, unique to TDPV
TDPV-107	78584-77778	268	FeP2 071	268	FGPV 068	virion protein	97.40%	I

TDPV-108	78680-79501	273	FeP2 072	273	FeP2 072	T10 protein	97.44%	I
TDPV-109	79648-79517	43	FeP2 073	43	FeP2 073	hypothetical protein	100.00%	I
TDPV-110	80766-79903	287	FeP2 074	287	FeP2 074	hypothetical protein	98.61%	I
TDPV-111	80949-80785	54	FeP2 075	54	CPPV 113	hypothetical protein	93.55%	I
TDPV-112	81270-81022	82	FeP2 076	82	FWPV 072	beta-NGF-like family protein	88.75%	F/T
TDPV-113	81338-81433	31			FGPV 073	beta-NGF-like family protein	81.82%	F/T
TDPV-114	81522-81430	30			FGPV 073	beta-NGF-like family protein	88.00%	F/T
TDPV-115	81827-81735	30	FeP2 077	106	FGPV 074	IL-18 binding protein	96.15%	F/T
TDPV-116	82046-81879	55	FeP2 077	106	FGPV 074	IL-18 binding protein	75.41%	F/T
TDPV-117	82263-82105	52			CPPV 116	hypothetical protein	85.11%	F/T
TDPV-118	82538-82299	79			CPPV 116	hypothetical protein	87.30%	F/T
TDPV-119	82886-82584	100	FeP2 078	101	FeP2 078	hypothetical protein	98.00%	I
TDPV-120	83453-82890	187	FeP2 079	187	FeP2 079	N1R|p28 family protein	97.86%	I
TDPV-121	83658-83533	41			CPPV 119	beta-NGF-like family protein	82.61%	F/T
TDPV-122	84012-84389	125	FeP2 080	125	PEPV 081	glutaredoxin	100.00%	I
TDPV-123	85039-84362	225	FeP2 081	225	FeP2 081	putative elongation factor	100.00%	I
TDPV-124	85033-85344	103	FeP2 082	103	PEPV 083	hypothetical protein	97.56%	I
TDPV-125	86379-85372	335	FeP2 083	335	FeP2 083	transforming growth factor B	97.31%	I
TDPV-126	86450-88336	628	FeP2 084	627	PPV 082	metalloprotease	98.57%	I
TDPV-127	90368-88320	682	FeP2 085	682	FeP2 085	DNA|RNA helicase|NPH-11	99.27%	I
TDPV-128	90401-91666	421	FeP2 086	421	FeP2 086	virion core proteinase	98.81%	I
TDPV-129	91669-92844	391	FeP2 087	391	FeP2 087	DNA-binding protein	98.98%	I
TDPV-130	92845-93090	81	FeP2 088	81	PEPV 089	IMV membrane protein	98.77%	I
TDPV-131	93100-93651	183	FeP2 089	183	FeP2 089	thymidine kinase	98.91%	I
TDPV-132	93717-93992	91	FeP2 090	91	PEPV 091	HT motif family protein	96.70%	I
TDPV-133	94050-94898	282	FeP2 091	291	FeP2 091	DNA-binding phosphoprotein	97.52%	I
TDPV-134	94899-95096	65	FeP2 092	65	FeP2 092	hypothetical protein	98.46%	I
TDPV-135	95103-96038	311	FeP2 093	311	FeP2 093	virion protein	99.36%	I
TDPV-136	96039-96143	34	FeP2 094	34	FGPV 092	MV entry/fusion complex protein	97.06%	I
TDPV-137	96207-98177	656	FeP2 095	656	FeP2 095	hypothetical protein	99.09%	I
TDPV-138	98119-98514	131	FeP2 096	131	FeP2 096	hypothetical protein	98.47%	I
TDPV-139	98795-98511	94	FeP2 097	94	FeP2 097	sulfhydryl oxidase ERV1	98.94%	I
TDPV-140	98822-101788	988	FeP2 098	988	FeP2 098	DNA polymerase	99.09%	I
TDPV-141	102622-101780	280	FeP2 099	282	FeP2 099	hypothetical protein	96.45%	I
TDPV-142	104330-102615	571	FeP2 100	571	FGPV 098	hypothetical protein	98.60%	I
TDPV-143	110164-104441	1907	FeP2 101	1885	PEPV 102	B22R family protein	98.88%	I
TDPV-144	110170-110265	31			CPPV 144	poly(A) polymerase large subunit PAPL	93.55%	I
TDPV-145	115611-110230	1793	FeP2 102	1826	FeP2 102	B22R family protein	97.74%	I

TDPV-146	115601-115696	31			unique			hypothetical protein, unique to TDPV
TDPV-147	121624-115853	1923	FeP2 103	1937	FeP2 103	B22R family protein	97.26%	I
TDPV-148	121693-122241	182	FeP2 104	182	FeP2 104	RNA polymerase subunit RPO30	98.90%	I
TDPV-149	122294-124447	717	FeP2 105	717	FeP2 105	hypothetical protein	98.33%	I
TDPV-150	124434-125852	472	FeP2 106	472	FeP2 106	polyA polymerase large subunit PAP-L	98.94%	I
TDPV-151	126190-125846	114	FeP2 107	114	FWPV 103	DNA-binding virion core phosphoprotein	100.00%	I
TDPV-152	126267-126899	210	FeP2 108	210	FeP2 108	hypothetical protein	96.19%	I
TDPV-153	127019-127468	149	FeP2 109	149	FeP2 109	hypothetical protein	99.33%	I
TDPV-154	127671-127970	99	FeP2 110	99	FeP2 110	hypothetical protein	95.96%	I
TDPV-155	133367-128025	1780	FeP2 111	1780	PPV 109	B22R family protein	98.88%	I
TDPV-156	133530-133438	30			unique			hypothetical protein, unique to TDPV
TDPV-157	133552-134685	377	FeP2 112	377	FeP2 112	virion envelope protein	98.94%	I
TDPV-158	134721-136640	639	FeP2 113	639	FeP2 113	virion release protein	98.75%	I
TDPV-159	136685-138043	452	FeP2 114	411	CPPV 160	hypothetical protein	95.80%	I
TDPV-160	138121-139455	444	FeP2 115	444	FeP2 115	SER|THR protein kinase	98.87%	I
TDPV-161	139430-140071	213	FeP2 116	213	FeP2 116	hypothetical protein	97.65%	I
TDPV-162	140344-140015	109			unique			hypothetical protein, unique to TDPV
TDPV-163	140674-140477	65			CPPV 164	virion core protein	94.12%	I
TDPV-164	140654-140851	65			CPPV 165	hypothetical protein	93.94%	I
TDPV-165	140844-141395	183	FeP2 118	183	PPV 116	HAL3 domain protein	100.00%	I
TDPV-166	141471-141737	88			CPPV 167	ankyrin repeat protein	91.36%	F/T
TDPV-167	141843-142469	208			FGPV 117	ankyrin repeat family protein	77.63%	I
TDPV-168	142555-142662	35			unique			hypothetical protein, unique to TDPV
TDPV-169	143071-143439	122	FeP2 121	122	FeP2 121	CC chemokine family protein	99.18%	I
TDPV-170	143555-143442	37			unique			hypothetical protein, unique to TDPV
TDPV-171	143617-143736	39			unique			hypothetical protein, unique to TDPV
TDPV-172	143913-145235	440	FeP2 122	440	FeP2 122	hypothetical protein	98.86%	I
TDPV-173	145237-145428	63	FeP2 123	63	PEPV 125	RNA polymerase subunit RPO7	100.00%	I
TDPV-174	145428-145994	188	FeP2 124	188	FGPV 123	hypothetical protein	99.47%	I
TDPV-175	146990-145959	343	FeP2 125	343	FeP2 125	virion core protein	99.42%	I
TDPV-176	147243-147151	30			unique			hypothetical protein, unique to TDPV
TDPV-177	147665-148267	200	FeP2 126	203	FeP2 126	hypothetical protein	95.57%	I
TDPV-178	148397-148522	41			unique			hypothetical protein, unique to TDPV
TDPV-179	148602-149321	239	FeP2 127	239	FeP2 127	hypothetical protein	98.33%	I
TDPV-180	149907-150542	211	FeP2 128	211	FeP2 128	thymidylate kinase	98.58%	I
TDPV-181	150597-151379	260	FeP2 129	260	PEPV 136	VLTF-1	100.00%	I
TDPV-182	151392-152402	336	FeP2 130	336	PPV 128	myristylated protein	99.68%	I
TDPV-183	152403-153134	243	FeP2 131	243	FeP2 131	myristylated protein	99.18%	I

TDPV-184	153169-153459	96	FeP2 132	96	FeP2 132	hypothetical protein	100.00%	I
TDPV-185	154354-153449	301	FeP2 133	301	FeP2 133	hypothetical protein	99.67%	I
TDPV-186	154380-155141	253	FeP2 134	253	FeP2 134	DNA-binding virion core VP8	98.81%	I
TDPV-187	155142-155531	129	FeP2 135	129	FeP2 135	hypothetical protein	100.00%	I
TDPV-188	155482-155928	148	FeP2 136	148	FGPV 139	hypothetical protein	100.00%	I
TDPV-189	155961-156887	308	FeP2 137	308	FeP2 137	polyA polymerase PAPs	99.03%	I
TDPV-190	156884-157444	186	FeP2 138	186	FeP2 138	RNA polymerase subunit RPO22	99.46%	I
TDPV-191	157847-157434	137	FeP2 139	137	FeP2 139	membrane protein	98.54%	I
TDPV-192	157888-161751	1287	FeP2 140	1287	FeP2 140	RNA polymerase subunit RPO147	99.22%	I
TDPV-193	162257-161757	166	FeP2 141	166	FeP2 141	protein tyrosine phosphatase	99.40%	I
TDPV-194	162273-162845	190	FeP2 142	190	FeP2 142	hypothetical protein	99.47%	I
TDPV-195	164027-163026	333	FeP2 143	333	FWPV 140	IMV envelope protein	100.00%	I
TDPV-196	166427-164028	799	FeP2 144	799	FeP2 144	RNA polymerase-associated protein RAP94	98.87%	I
TDPV-197	166577-167101	174	FeP2 145	174	FeP2 145	VLTF-4	98.77%	I
TDPV-198	167102-168052	316	FeP2 146	316	FeP2 146	DNA topoisomerase	98.73%	I
TDPV-199	168057-168521	154	FeP2 147	152	PEPV 154	putative 17kDa protein	98.05%	I
TDPV-200	168795-168484	103	FeP2 148	103	FeP2 148	hypothetical protein	96.43%	I
TDPV-201	168803-171361	852	FeP2 149	852	FeP2 149	mRNA capping enzyme, large subunit	99.30%	I
TDPV-202	171339-171551	70	FeP2 150	63	FGPV 153	HT motif family protein	84.38%	I
TDPV-203	172120-171698	140	FeP2 151	140	FeP2 151	virion protein	97.86%	I
TDPV-204	172188-172322	44	FeP2 152	45	FeP2 152	hypothetical protein	93.33%	I
TDPV-205	172471-173043	190	FeP2 153	190	FeP2 153	hypothetical protein	100.00%	I
TDPV-206	173111-173977	288	FeP2 154	284	FeP2 154	N1R|p28 family protein	96.53%	I
TDPV-207	174019-174732	237	FeP2 155	238	FeP2 155	Dck	98.32%	I
TDPV-208	175128-174742	128	FeP2 158	132	FGPV 158	HT motif family protein	95.31%	I
TDPV-209	175219-175848	209	FeP2 156	209	FeP2 156	hypothetical protein	98.56%	I
TDPV-210	176017-176187	56			CPPV 218	N1R/p28 family protein	84.21%	I
TDPV-211	176350-176505	51			PEPV 163	hypothetical protein	80.56%	I
TDPV-212	176578-177810	410	FeP2 157	410	FeP2 157	N1R|p28 family protein	97.80%	I
TDPV-213	177859-178020	53			CPPV 220	hypothetical protein	96.15%	I
TDPV-214	178054-178452	132	FeP2 158	132	FeP2 158	HT motif family protein	99.24%	I
TDPV-215	178498-179481	327	FeP2 159	327	FeP2 159	N1R|p28 family protein	99.69%	I
TDPV-216	179536-180930	464	FeP2 160	464	FeP2 160	photolyase	99.14%	I
TDPV-217	180875-181015	46			CPPV 224	hypothetical protein	89.13%	I
TDPV-218	181068-181808	246	FeP2 161	246	CPPV 225	N1R|p28 family protein	98.37%	I
TDPV-219	181889-182359	156	FeP2 162	156	FeP2 162	hypothetical protein	100.00%	I
TDPV-220	182407-182856	149	FeP2 163	149	FeP2 163	N1R|p28 family protein	97.99%	I

TDPV-221	182890-183291	133	FeP2 164	133	FeP2 164	N1R|p28 family protein	98.50%	I
TDPV-222	183341-183478	45	FeP2 165	45	FeP2 165	hypothetical protein	100.00%	I
TDPV-223	183494-183979	161	FeP2 166	160	FeP2 166	N1R|p28 family protein	98.76%	I
TDPV-224	184142-184005	45			unique			hypothetical protein, unique to TDPV
TDPV-225	184463-184287	58			unique			hypothetical protein, unique to TDPV
TDPV-226	184411-184566	51	FeP2 167	591	FeP2 167	ankyrin repeat protein	89.13%	I
TDPV-227	184870-184601	89			unique		93.18%	hypothetical protein, unique to TDPV
TDPV-228	184677-184985	102	FeP2 167	591	FeP2 167	ankyrin repeat protein	90.00%	I
TDPV-229	185094-185393	99	FeP2 167	591	FeP2 167	ankyrin repeat protein	95.88%	I
TDPV-230	186260-186114	48			unique			hypothetical protein, unique to TDPV
TDPV-231	186238-187008	256	FeP2 168	256	FeP2 168	N1R|p28 family protein	96.88%	I
TDPV-232	187121-186954	55			unique			hypothetical protein, unique to TDPV
TDPV-233	187133-187273	46			FGPV 172	ankyrin repeat family protein	94.44%	F/T
TDPV-234	187465-187352	37			FGPV 172	aankyrin repeat family protein	91.18%	F/T
TDPV-235	187471-187677	68			FGPV 172	ankyrin repeat family protein	98.15%	F/T
TDPV-236	187683-187901	72			PEPV 177	ankyrin repeat protein	85.45%	F/T
TDPV-237	188098-188361	87			FGPV 172	ankyrin repeat family protein	80.00%	F/T
TDPV-238	188443-188348	31			FGPV 172	ankyrin repeat family protein	93.55%	F/T
TDPV-239	188884-189090	68	FeP2 169	46	FGPV 172	ankyrin repeat family protein	94.00%	F/T
TDPV-240	189267-190427	386	FeP2 170	383	FeP2 170	hypothetical protein	96.63%	I
TDPV-241	190607-190837	76			unique			hypothetical protein, unique to TDPV
TDPV-242	190845-191021	58			unique			hypothetical protein, unique to TDPV
TDPV-243	191209-191102	35			unique			hypothetical protein, unique to TDPV
TDPV-244	191236-191355	39			unique			hypothetical protein, unique to TDPV
TDPV-245	192315-191638	225	FeP2 171	225	FeP2 171	late transcription factor VLTF-3	99.11%	I
TDPV-246	192530-192312	72	FeP2 172	72	PEPV 181	virus redox protein	100.00%	I
TDPV-247	194531-192546	661	FeP2 173	658	FeP2 173	virion core protein P4b	98.64%	I
TDPV-248	195411-194614	265	FeP2 174	244	FGPV 181	immunodominant virion protein	96.47%	I
TDPV-249	195450-195959	169	FeP2 175	169	FeP2 175	RNA polymerase subunit RPO19	100.00%	I
TDPV-250	197078-195954	374	FeP2 176	374	FeP2 176	hypothetical protein	98.93%	I
TDPV-251	199214-197085	709	FeP2 177	709	FeP2 177	early transcription factor large subunit VETF-L	98.73%	I
TDPV-252	199280-200185	301	FeP2 178	301	FeP2 178	intermediate transcription factor VITF-3	99.00%	I
TDPV-253	200377-200147	76	FeP2 179	76	FeP2 179	hypothetical protein	100.00%	I
TDPV-254	203053-200378	891	FeP2 180	891	FeP2 180	virion core protein P4a	99.55%	I
TDPV-255	203071-203889	272	FeP2 181	272	PEPV 190	hypothetical protein	98.90%	I
TDPV-256	204417-203890	175	FeP2 182	175	FeP2 182	virion protein	99.38%	I
TDPV-257	204432-204545	37	FeP2 183	49	PEPV 192	hypothetical protein	100.00%	I

TDPV-258	204840-204625	71	FeP2 184	71	FeP2 184	virion protein	98.59%	I
TDPV-259	205182-204907	91	FeP2 185	91	FeP2 185	virion envelope protein	100.00%	I
TDPV-260	205360-205199	53	FeP2 186	53	PEPV 195	virion envelope protein	100.00%	I
TDPV-261	205669-205376	97	FeP2 187	91	FeP2 187	hypothetical protein	100.00%	I
TDPV-262	206762-205653	369	FeP2 188	369	FeP2 188	putative mystirilated membrane protein	99.46%	I
TDPV-263	207374-206778	198	FeP2 189	198	FeP2 189	phosphorylated virion membrane protein	99.49%	I
TDPV-264	207392-208780	462	FeP2 190	462	FeP2 190	DNA helicase	98.70%	I
TDPV-265	209014-208748	88	FeP2 191	88	FeP2 191	hypothetical protein	97.73%	I
TDPV-266	209363-209022	113	FeP2 192	113	FeP2 192	hypothetical protein	99.12%	I
TDPV-267	209362-210663	433	FeP2 193	432	FeP2 193	processivity factor	98.15%	I
TDPV-268	210663-211133	156	FeP2 194	156	FeP2 194	hypothetical protein	98.72%	I
TDPV-269	211143-212297	384	FeP2 195	383	FeP2 195	intermediate transcription factor VITF-3	95.31%	I
TDPV-270	212324-215797	1157	FeP2 196	1157	FeP2 196	RNA polymerase subunit RPO132	99.31%	I
TDPV-271	217615-215786	609	FeP2 197	608	FeP2 197	A-type inclusion protein	97.87%	I
TDPV-272	219075-217651	474	FeP2 198	472	FeP2 198	A-type inclusion protein	96.20%	I
TDPV-273	219498-219076	140	FeP2 199	140	PEPV 208	hypothetical protein	99.29%	I
TDPV-274	220421-219513	302	FeP2 200	302	PEPV 209	RNA polymerase subunit RPO35	98.01%	I
TDPV-275	220620-220396	74	FeP2 201	74	FeP2 201	hypothetical protein	100.00%	I
TDPV-276	220789-220676	37	FeP2 202	37	PEPV 211	A30.5L-like protein	100.00%	I
TDPV-277	220799-221140	113	FeP2 203	113	FeP2 203	hypothetical protein	100.00%	I
TDPV-278	221141-221503	120	FeP2 204	120	FeP2 204	hypothetical protein	93.33%	I
TDPV-279	222406-221492	304	FeP2 205	305	FGPV 212	virion assembly protein	97.05%	I
TDPV-280	222581-223102	173	FeP2 206	173	FeP2 206	C-type lectin-like protein	99.42%	I
TDPV-281	223161-223823	220	FeP2 207	106	PEPV 216	V-type Ig domain protein	99.09%	I
TDPV-282	223801-224592	263	FeP2 209	58	PEPV 217	V-type Ig domain protein	98.48%	I
TDPV-283	224631-225464	277	FeP2 210	277	FeP2 210	hypothetical protein	100.00%	I
TDPV-284	225519-226376	285	FeP2 211	285	FeP2 211	tyrosine protein kinase	98.60%	I
TDPV-285	226415-227443	342	FeP2 212	342	FeP2 212	serpin family protein	99.42%	I
TDPV-286	228109-227447	220	FeP2 213	220	PEPV 221	hypothetical protein	99.52%	I
TDPV-287	228224-229150	308	FeP2 214	308	CPPV 285	G-protein-coupled receptor family protein	98.70%	I
TDPV-288	229162-229464	100	FeP2 215	92	FeP2 215	hypothetical protein	87.00%	E
TDPV-289	229471-229722	83	FeP2 216	67	FGPV 222	beta-NGF-like family protein	86.00%	F/T
TDPV-290	229873-230022	49			CPPV 287	beta-NGF-like family protein	78.26%	F/T
TDPV-291	230138-230263	41			FGPV 223	HT motif family protein	77.78%	I
TDPV-292	230341-230922	193	FeP2 217	193	FeP2 217	hypothetical protein	97.93%	I
TDPV-293	231333-230914	139	FeP2 218	129	FeP2 218	HT motif family protein	96.15%	I
TDPV-294	231418-231561	47			FGPV 226	CC-chemokine family protein	80.00%	F/T
TDPV-295	231565-231696	43			FGPV 226	CC-chemokine family protein	77.50%	F/T

TDPV-296	231932-231801	43			CPPV 292	putative interleukin binding protein	93.10%	F/T
TDPV-297	231970-232209	79			CPPV 292	putative interleukin binding protein	85.29%	F/T
TDPV-298	232304-232669	121	FeP2 219	123	FeP2 219	epidermal growth factor-like protein	96.75%	I
TDPV-299	232672-233583	303	FeP2 220	303	FeP2 220	serine|threonine protein kinase	98.02%	I
TDPV-300	233678-234043	121	FeP2 221	162	FeP2 221	hypothetical protein	96.64%	F/T
TDPV-301	234177-234554	125	FeP2 222	125	PPV 219	hypothetical protein	95.20%	I
TDPV-302	234653-234877	74	FeP2 223	74	PEPV 232	hypothetical protein	100.00%	I
TDPV-303	235053-235145	30	FeP2 224	173	CPPV 300	hypothetical protein	92.59%	F/T
TDPV-304	235266-235156	36			CPPV 300	hypothetical protein	94.44%	F/T
TDPV-305	235326-235475	49	FeP2 224	173	CPPV 300	hypothetical protein	91.18%	F/T
TDPV-306	235601-236485	294	FeP2 225	294	CPPV 301	ankyrin repeat protein	94.93%	I
TDPV-307	236536-236991	151	FeP2 226	143	FeP2 226	host range protein	99.30%	E
TDPV-308	237137-237232	31			CPPV 303	hypothetical protein	94.74%	F/T
TDPV-309	237508-237609	33			CPPV 303	hypothetical protein	91.30%	F/T
TDPV-310	237866-237756	36			CPPV 303	hypothetical protein	85.71%	F/T
TDPV-311	238527-238805	92	FeP2 230	182	FGPV 237	ankyrin repeat protein	94.44%	F/T
TDPV-312	238825-239085	86	FeP2 230	182	CPPV 305	ankyrin repeat protein	87.91%	F/T
TDPV-313	239280-240602	440	FeP2 231	440	FeP2 231	ankyrin repeat protein	97.73%	I
TDPV-314	241155-240604	183	FeP2 232	183	FeP2 232	A47L-like protein	99.45%	I
TDPV-315	241249-243492	747	FeP2 233	747	FeP2 233	ankyrin repeat protein	98.13%	I
TDPV-316	243547-243693	48			CPPV 309	ankyrin repeat containing protein	83.72%	F/T
TDPV-317	244147-244254	35			unique			hypothetical protein, unique to TDPV
TDPV-318	244778-244918	46			CPPV 309	ankyrin repeat containing protein	97.22%	F/T
TDPV-319	245121-245999	292	FeP2 236	293	FeP2 236	serine|threonine protein kinase	98.98%	I
TDPV-320	246068-247153	361	FeP2 237	361	FeP2 237	ankyrin repeat protein	98.34%	I
TDPV-321	247280-247543	87			CPPV 312	ankyrin repeat protein	85.39%	F/T
TDPV-322	247807-248355	182	FeP2 239	39	CPPV 312	ankyrin repeat protein	94.67%	F/T
TDPV-323	248821-248531	96			CPPV 312	ankyrin repeat protein	89.13%	F/T
TDPV-324	248787-249746	319	FeP2 241	319	CPPV 313	ankyrin repeat protein	90.88%	I
TDPV-325	250184-250894	236	FeP2 242	503	FeP2 242	ankyrin repeat protein	96.12%	F/T
TDPV-326	250971-251294	107	FeP2 242	503	FeP2 242	ankyrin repeat protein	97.20%	F/T
TDPV-327	251542-251637	31			unique			hypothetical protein, unique to TDPV
TDPV-328	252050-251934	38			FGPV 248	serpin family protein	84.00%	F/T
TDPV-329	252127-252249	40			FGPV 248	serpin family protein	86.96%	F/T
TDPV-330	252330-252220	36			FGPV 248	serpin family protein	73.08%	F/T
TDPV-331	252881-252324	185	FeP2 243	185	FeP2 243	putative A47L-like protein	99.46%	I
TDPV-332	252956-253291	111	FeP2 244	51	PEPV 251	ankyrin repeat protein	91.09%	F/T
TDPV-333	253275-254375	366	FeP2 245	410	CPPV 317	ankyrin repeat protein	94.21%	F/T

TDPV-334	254570-256015	481	FeP2 247	481	FeP2 247	ankyrin repeat protein	97.92%	I
TDPV-335	256059-257567	502	FeP2 248	502	FeP2 248	ankyrin repeat protein	97.01%	I
TDPV-336	257621-258898	425	FeP2 249	80	CPPV 320	ankyrin repeat protein	90.54%	I
TDPV-337	259285-260124	279	FeP2 252	279	FeP2 252	N1R|p28 family protein	97.13%	I
TDPV-338	260151-259972	59	FeP2 253	122	FeP2 253	hypothetical protein	96.49%	F/T
TDPV-339	260206-260415	69			CPPV 323	putative P-type ATPase	80.88%	I
TDPV-340	260533-260432	33			unique			hypothetical protein, unique to TDPV
TDPV-341	260600-260499	33			FGPV 257	C-type lectin family protein	83.33%	F/T
TDPV-342	260825-260646	59	FeP2 254	72	PEPV 258	C-type lectin family protein	74.55%	F/T
TDPV-343	260969-262204	411	FeP2 255	411	FeP2 255	ankyrin repeat protein	96.59%	I
TDPV-344	262227-262406	59			CPPV 326	hypothetical protein	87.27%	I
TDPV-345	262431-262844	137			unique			hypothetical protein, unique to TDPV
TDPV-346	263071-263271	66			PEPV 261	ankyrin repeat protein	92.42%	F/T
TDPV-347	263396-263268	42			FGPV 260	ankyrin repeat protein	86.49%	F/T
TDPV-348	263353-263481	42			unique			hypothetical protein, unique to TDPV
TDPV-349	263846-263971	41			unique			hypothetical protein, unique to TDPV
TDPV-350	263968-264081	37			unique			hypothetical protein, unique to TDPV
TDPV-351	264036-264188	50			FWPV 246	ankyrin repeat protein	51.22%	F/T
TDPV-352	264204-264419	71			CPPV 328	hypothetical protein	93.10%	F/T
TDPV-353	264433-265065	210	FeP2 256	209	FeP2 256	Ig domain protein	97.62%	I
TDPV-354	265116-265409	97	FeP2 257	628	FeP2 257	ankyrin repeat protein	96.91%	F/T
TDPV-355	265450-266970	506	FeP2 257	628	FeP2 257	ankyrin repeat protein	99.01%	F/T
TDPV-356	267012-267590	192	FeP2 258	192	FeP2 258	ankyrin repeat protein	98.44%	I
TDPV-357	267692-267970	92			FGPV 267	V-type Ig domain protein	81.71%	F/T
TDPV-358	268103-267996	35			FGPV 267	V-type Ig domain protein	95.65%	F/T
TDPV-359	268309-268184	41			FGPV 267	V-type Ig domain protein	74.07%	F/T
TDPV-360	268481-268615	44	FeP2 259	54	FGPV 267	V-type Ig domain protein	100.00%	F/T
TDPV-361	268576-268725	49	FeP2 259	54	FGPV 267	V-type Ig domain protein	100.00%	F/T
TDPV-362	268981-270984	667	FeP2 260	667	FeP2 260	hypothetical protein	98.20%	I
TDPV-363	271258-271115	47	FeP2 261	75	FGPV 269	ankyrin repeat protein	87.50%	F/T
TDPV-364	272037-272213	58	FeP2 262	227	CPPV 335	Ig-like domain protein	94.34%	F/T
TDPV-365	272512-272384	42			CPPV 335	Ig-like domain protein	93.02%	F/T
TDPV-366	272483-272683	66	FeP2 262	227	CPPV 335	Ig-like domain protein	93.33%	F/T
TDPV-367	272755-273462	235	FeP2 263	235	CPPV 336	Immunoglobulin-like domain protein	92.34%	I
TDPV-368	273558-275294	578	FeP2 264	585	PEPV 272	ankyrin repeat protein	94.86%	I
TDPV-369	275375-275749	124	FeP2 265	122	CPPV 339	Efc family protein	97.58%	I
TDPV-370	275866-276315	149	FeP2 266	149	FeP2 266	N1R|p28 family protein	98.66%	I
TDPV-371	276492-276388	34			unique			hypothetical protein, unique to TDPV

TDPV-372	277532-277855	107	FeP2 267	107	FeP2 267	hypothetical protein	96.26%	I
TDPV-373	277924-278298	124	FeP2 268	124	FeP2 268	C-type lectin-like protein	95.97%	I
TDPV-374	278568-278323	81			CPPV 006	hypothetical protein	91.53%	F/T
TDPV-375	278768-278553	71			CPPV 006	hypothetical protein	91.55%	F/T
TDPV-376	279454-278786	222	FeP2 269	222	PEPV 004	hypothetical protein	95.50%	F/T
TDPV-377	279641-279775	44			unique			hypothetical protein, unique to TDPV
TDPV-378	280166-280771	201	FeP2 271	200	FeP2 001	C-type lectin-like protein	91.54%	I
TDPV-379	280912-281100	62			unique			hypothetical protein, unique to TDPV
TDPV-380	281189-281290	33			unique			hypothetical protein, unique to TDPV

Note: FeP2, Pigeonpox virus; PPV, Pigeonpox virus; FGPV, Flamingopox virus; CPPV, Cook's petrelpox virus; PEPV, Penguinpox virus; FWPV, Fowlpox virus. F/T: Fragment/Truncated, the length of the ORF is <80% of the closest homologue. I: Intact, the 5′ end of the ORF is intact and the length of the ORF is ≥80% of the closest homologue. E: Extended, if the 5′ and 3′ end of the ORF is intact or as extended at the 5′ and 3′ end, while the length of the ORF is >20% of the closest homologue.
